# Functional Skin Grafts: Where Biomaterials Meet Stem Cells

**DOI:** 10.1155/2019/1286054

**Published:** 2019-07-01

**Authors:** Amtoj Kaur, Swati Midha, Shibashish Giri, Sujata Mohanty

**Affiliations:** ^1^Stem Cell Facility (DBT-Centre of Excellence for Stem Cell Research), All India Institute of Medical Sciences, New Delhi, India; ^2^Department of Cell Techniques and Applied Stem Cell Biology, Centre for Biotechnology and Biomedicine, University of Leipzig, Deutscher Platz 5, D-04103 Leipzig, Germany; ^3^Department of Plastic Surgery and Hand Surgery, University Hospital Rechts der Isar, Technische Universität München, Munich, Germany

## Abstract

Skin tissue engineering has attained several clinical milestones making remarkable progress over the past decades. Skin is inhabited by a plethora of cells spatiotemporally arranged in a 3-dimensional (3D) matrix, creating a complex microenvironment of cell-matrix interactions. This complexity makes it difficult to mimic the native skin structure using conventional tissue engineering approaches. With the advent of newer fabrication strategies, the field is evolving rapidly. However, there is still a long way before an artificial skin substitute can fully mimic the functions and anatomical hierarchy of native human skin. The current focus of skin tissue engineers is primarily to develop a 3D construct that maintains the functionality of cultured cells in a guided manner over a period of time. While several natural and synthetic biopolymers have been translated, only partial clinical success is attained so far. Key challenges include the hierarchical complexity of skin anatomy; compositional mismatch in terms of material properties (stiffness, roughness, wettability) and degradation rate; biological complications like varied cell numbers, cell types, matrix gradients in each layer, varied immune responses, and varied methods of fabrication. In addition, with newer biomaterials being adopted for fabricating patient-specific skin substitutes, issues related to escalating processing costs, scalability, and stability of the constructs under *in vivo* conditions have raised some concerns. This review provides an overview of the field of skin regenerative medicine, existing clinical therapies, and limitations of the current techniques. We have further elaborated on the upcoming tissue engineering strategies that may serve as promising alternatives for generating functional skin substitutes, the pros and cons associated with each technique, and scope of their translational potential in the treatment of chronic skin ailments.

## 1. Introduction

Skin, the largest organ of the human body, acts as a barrier for outside pollutants and microbes; hence, serving as the body's first line of defense. In addition, skin performs various functions like thermoregulation, moisture retention, immune protection, imparting sensation, and self-healing response [1–3]. The human skin comprises of three layers: epidermis (outermost), dermis (middle), and hypodermis (deeper) [4]. The epidermis is a 0.2 mm thick, packed sheath of cells consisting of keratinocytes, which are in different stages of differentiation, along with melanocytes and epidermal stem cells confined to the basal proliferative layer. Furthermore, there are 4 layers within the epidermis, namely, the stratum corneum (dead cornified layer with 15-30 sheets of corneocytes), stratum granulosum (3-5 sheets of flattened keratinocytes with arrested division), stratum spinosum (possessing 8-10 layers of keratinocytes with restricted cell division), and stratum basale (proliferative layer). The “bricks-and-mortar” array type of organization of corneocytes in the epidermis acts as a barrier separating the internal body environment from the external along with regulating fluid loss [5]. The dermis, comprising of a thick connective tissue, is sandwiched in the middle of the epidermis and the hypodermis [6]. It is constituted of a bed of glycosaminoglycans (GAGs), elastin, and collagen extracellular matrix (ECM) with embedded fibroblasts. It also possesses numerous skin appendages like sebaceous and sweat glands, mechanoreceptors, hair follicles, vasculature, and nerve endings. The dermis imparts sensory and mechanical properties to the skin. A separating layer of basement membrane having a specialized ECM composition (constituting of collagens III, IV, and VII; laminins; and fibrillin) is present between the epidermis and dermis facilitating diffusion and communication between the cells *via* paracrine signaling to maintain homeostasis [7, 8]. The bottom-most hypodermis or subcutaneous layer comprises of adipose tissue and controls the mechanical and thermoregulatory properties of the skin.

Burns, acute trauma, chronic wounds, intensive surgeries, infections, and genetic abnormalities are the most common factors responsible for causing variable extents of damage to the skin [9–11]. According to the World Health Organization (WHO), fatal injuries arising from burns account for approximately 180,000 deaths annually. In India alone, over 1,000,000 burn victims suffer from moderate to serious burns per annum. The global wound care market is expected to increase from 18.35 billion USD in 2017 to 22.81 billion USD by 2022 [12]. Apart from the huge cost of treatment, indirect expenses such as lost income due to unemployment, prolonged medical care, and emotional trauma immensely contribute to the socioeconomic impact. Wounds act as breaches in the tissue which compromise the defensive ability of the skin; hence, becoming the leading cause of infections. Based on the depth of injury, skin wounds have four subdivisions: (i) epidermal (top layer of skin), (ii) superficial partial thickness (epidermis and upper dermis), (iii) deep partial thickness (epidermis and full dermis), and (iv) full thickness (all three layers of the skin) [13]. In the case of deeper skin injuries including partial and full-thickness wounds, the natural healing mechanism is incapable of restoring the fully functional tissue in most cases [14], except where hair follicles are present. Therefore, skin wound healing poses a serious challenge for both patients and plastic surgeons.

**Figure 1:**
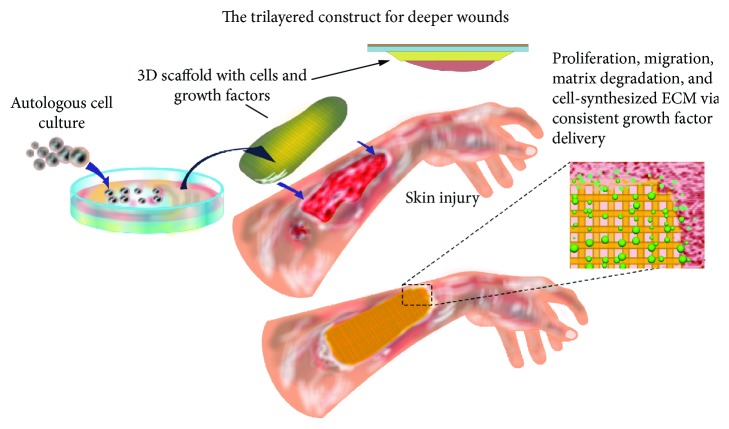
Schematic illustrating the stages of skin tissue engineering using biomaterials and stem cell technology. Briefly, autologous cells are isolated from skin biopsies of patients and expanded *in vitro* for up to 3 weeks. When optimal cell confluency is achieved, cells in combination with growth- and differentiation-inducing factors are seeded on biomimetic scaffolds (with structural resemblance to the skin anatomy) for implantation into the target site to facilitate repair and regeneration of the damaged skin tissue.

Since their origin in 1874, autologous split-thickness skin grafts (STSG) have been considered as the “gold standards” for treating skin injuries requiring ample amount of healthy skin [15]. STSG aids in the transfer of epidermal stem cells from a healthy site to the wound site. However, this approach faces drawbacks related to donor site shortage, failure to treat full-thickness wounds, and scarring at both the donor and recipient sites [16]. Moreover, the increasing gap between the demand and supply of autologous and allogenic grafts has paved the way for skin tissue engineering (STE). STE takes advantage of an artificial construct where autologous cells of the individual are isolated, cultured on constructs, and implanted into the wound site to facilitate the healing process (Figure 1). From the pioneering work performed by Jacques-Louis Reverdin in 1870 by the application of “fresh skin” allografts, skin replacement and regenerative therapy has now come a long way utilizing different biological materials with cultured cells in modern medicine [17]. Today, the broad realm of STE covers numerous cutting-edge strategies such as nanotechnology, 3D bioprinting, stem cells, and microfluidics [18–20]. There are some key features that are crucial for tissue-engineered skin substitutes such as biocompatibility, nontoxicity, nonimmunogenicity, biodegradability, moisture retention property, optimal elasticity, and porosity with good interconnectivity for a free exchange of gases and nutrients to induce the growth of neovasculature for generating functional skin substitutes. In order to attain commercial relevance, these engineered skin substitutes must be cost-effective, scalable, have a prolonged shelf life, and should be available off-the-shelf for large-scale application [21–24].

In this review, we discuss the progress made so far in the development of artificial skin scaffolds using various innovative strategies and biomaterials for the fabrication of skin tissue substitutes, along with their clinical applications and future perspectives.

## 2. Scaffold Characteristics Specific to Skin

While several tissue-engineered scaffolds are being developed, each material needs to be modulated to be able to match the properties of the target tissue. Even after attaining considerable clinical success with the currently available commercial skin replacements, the search for an ideal, functional skin substitute, which exactly recapitulates the patient's original tissue, remains elusive. Some key challenges include; (i) selecting the most suitable biofabrication approach that can simulate the complex anatomical hierarchy of trilayered skin; (ii) optimizing multimaterial compositions with desired properties to aid in cellular guidance and differentiation to mimic the different layers; (iii) and determining the type and source of stem cells, seeding modality (single cell suspension, aggregates), and seeding density in terms of variability between the different layers.

### 2.1. Types of Skin Substitutes

Depending upon the depth of the tissue, skin substitutes can be categorized into four distinct types.

#### 2.1.1. Epidermal Skin Constructs

The epidermal skin constructs comprise of keratinocytes cultured on a layer of irradiated feeder cells of murine fibroblasts. The autologous keratinocytes isolated from the patient usually take 2-3 weeks in expansion media to develop cell sheets of stratified keratinocytes, commonly termed as cultured epithelial autografts (CEAs). CEAs are typically 2 to 8 layers thick. However, they are not very effective for curing burns and are fragile to handle [25]. Petroleum gauze dressings and silicone membranes have been used to render support to the mechanically inferior cell sheets. Acid functionalization performed on materials aids in the easy transfer of keratinocytes, apart from aiding in attachment and proliferation [26]. However, these synthetic carriers are nonbiodegradable and need to be removed after sometime. Therefore, a few studies have documented the usage of natural biomaterials such as fibrin [27, 28] and hyaluronic acid (HA) [29] as carriers for cultured keratinocytes as they provide a conducive microenvironmental niche for promoting migration, proliferation, matrix degradation, and differentiation of keratinocytes.

#### 2.1.2. Dermal Skin Constructs

Dermis comprises of ECM with fibroblasts [30] which is further divided into an upper “papillary” and lower “reticular” region. The conformational orientation of thin, randomly aligned collagen fiber bundles (primarily collagen type III) in the papillary region form an intricate ridge-like arrangement. The reticular dermis, on the other hand, is composed of a more ordered collagen arrangement of predominantly collagen type I. Most of the commercial dermal skin replacements are cell free and act as an initial framework for facilitating infiltration of cells and blood vessels from the host tissue. This is mainly due to low fabrication cost, easy storage, and low immunogenic response [31]. Fibroblasts have shown to repopulate acellular dermal substitutes *in vivo*, 7 days post implantation [32]. In contrast, in dermal skin substitutes with allogenic human neonatal fibroblasts like Apligarf, the cells did not survive beyond a few weeks post implantation [33].

#### 2.1.3. Epidermal-Dermal Skin Constructs

Currently, the closest and the most sophisticated skin biomimic available in the market is an epidermal-dermal skin substitute comprising of both of the upper layers of the skin. The close association between keratinocytes and fibroblasts in the epidermal-dermal skin grafts triggers a cascade of biological moieties (growth factors, cytokines) to expedite tissue healing [34–36]. Significant enhancement in wound closure has been observed where these epidermal-dermal skin constructs have been used to cure chronic injuries and ulcers [37]. Several attempts have been made using different fabrication techniques like electrospinning and 3D bioprinting to fabricate bilayered constructs [38, 39]. These bilayered constructs measure about 2.5 mm in thickness, which hinders adequate vascularization subsequently resulting in the early death of constructs. Hence, advancement in vascularization strategies is the prime requisite for developing functional bilayered skin constructs [40].

#### 2.1.4. Trilayered Skin Construct

Trilayered skin constructs include the hypodermal adipose tissue along with the dermis and the epidermis. It can be considered as the closest mimic to the native human skin for full-thickness wounds. The hypodermal layer consists of fatty connective tissue with predominantly collagen VI ECM and a multicellular organization (preadipocytes, adipocytes, vascular endothelial cells, and adipose macrophages). A few attempts have been made to fabricate trilayered skin constructs. Kober et al. fabricated a fibrin-based trilayered skin construct by depositing adipose-derived stem cells (ADSCs), fibroblasts, and keratinocytes in the fibrin matrix for replicating the hypodermis, dermis, and epidermis, respectively [41]. The fabricated construct showed a morphology similar to the native human skin. Another group used human plasma for a trilayered skin construct, engineered using a similar combination of cells [42]. There is a need for more extensive research in hypodermal engineering in order to cater to full-thickness wounds with special consideration to be given to zonal ECM variation present in the different layers of the skin.

### 2.2. Pigmentation

Pigmentation is not only an important cosmetic property of the skin, but melanin in the skin also protects against ultraviolet (UV) radiation. An off-the-shelf product, ReCell®, makes use of fresh skin biopsy to prepare a spray-on cell suspension comprising of a combination of autologous keratinocytes, melanocytes, and fibroblasts for treating vitiligo. In such grafts, repigmentation took approximately 3-5 weeks, while contrary reports showed delayed pigmentation which took as long as 4 months to set in [43, 44]. A limiting factor to the approach is the age dependency, as the product had limited efficacy (less than 65%) in patients >30 years of age [44]. Possible contributing factors could be the relatively thinner skin in elderly individuals [45]. Also, other factors for elderly patients may include compromised immune functions, disease condition, lower melanogenic capacity, and decreased vascularity with increasing age [46]. Apart from this, a 3D bioprinting approach has also been explored for the construction of pigmented skin constructs. Ng et al. demonstrated the use of a drop-on-demand bioprinting technique to bioprint a precise pattern of one melanocyte surrounded by 8 keratinocytes in a 3 × 3 array. The bioprinted skin appeared uniformly pigmented as compared to the manually casted construct after 39 days of *in vitro* culture [47].

### 2.3. Vasculature

Wound closure after full-thickness burns requires the reestablishment of a stable epidermis as a prerequisite. The stability of the epidermis depends upon the reformation of the basement membrane and vascularized connective tissues to anchor the outer skin to the body [48]. In skin constructs, anastomosis with the host vasculature is essential for the diffusion of oxygen, nutrients, and other biological moieties, as the diffusion limit is approximately 0.1-0.2 mm only [49]. Problems arise in the wound area primarily due to inadequate graft preparation, infection, and scarring of the tissue due to a hindered blood supply as a result of a long interval between injury and grafting (usually >3 days) [50]. Having an established vascular supply is critical in cases where the affected area is large. Few studies have developed prevascularized grafts and demonstrated the full-thickness healing of dermal wounds in preclinical models [51]. As opposed to this, pedicle flaps have been used clinically with the advantage being that they carry their own blood supply. For this to work, these blood supplies need to anastomose with the adjacent host tissue. But these flaps are much thicker than grafts and usually encounter problems with kinking of the matrix and delayed anastomosis [52]. To circumvent the problems associated with the current techniques, various approaches have been proposed to induce vasculature [53]. A blend of cellular [54], biomaterial-based [55–57], and microfabrication approaches [58] could possibly circumvent delayed vascularization at the site of injury. The addition of endothelial cells or stem/progenitor cells, a common cell-based approach, induces the formation of neovasculature at the injury site. Documented evidence dictates that the incorporation of hydrogels such as fibrin [55] or HA [56] within the bioengineered constructs can promote angiogenesis. In the microfabrication approach, sacrificial or nonsacrificial mini- and microchannels are created for the rapid diffusion of oxygen, nutrients, and growth factors. Detailed studies on various vascularization strategies have already been reviewed elsewhere [54, 59, 60].

### 2.4. Optimal Cell Source

A number of cell sources have been explored for STE. Embryonic stem cells have been isolated and differentiated into keratinocytes [61] and fibroblasts [62]. While embryonic stem cells (ESCs) can give rise to the most suitable differentiated cell population, their usage is restricted due to ethical concerns and their tendency to form teratomas. As an alternative, the dependency on cell lines increased considerably due to their robustness and immortalization. Different cell lines like the keratinocyte cell line (for example, HaCaT, immortalized adult skin keratinocyte) [63] and the fibroblast cell line (HFF, human foreskin fibroblast) [63] have been extensively used for STE. Being robust, the use of cell lines undoubtedly helps in better technique optimization but it fails to fully mimic the biological scenario as cell lines have altered properties which may lead to a discrepancy in the data and therefore are not very reliable. Also, they considerably differ from primary human cells in terms of high propensity and differential gene expression [64]. Autologous differentiated cells like keratinocytes, melanocytes, and fibroblasts are effective alternatives [39, 47]. Isolation of these differentiated autologous cells from various tissues and organs has been fully standardized [65–67]. Although, these cells provide the closest biological image of *in vivo* conditions, they are difficult to culture and handle, as they do not possess high proliferative capacity. This also leads to a need of high initial seeding density, which is usually difficult to get in cases of wounds and burns affecting large areas of the body. Therefore, several tissue sources are being explored to get easy access to primary cells. Bone marrow-derived mesenchymal stem cells (BM-MSCs) are the clinically proven cell source which finds wide-scale applications across different organs owing to their multilineage differentiation potential, but, their isolation procedure is very invasive. Another source of adult stem cells, called adipose-derived stem cells (ADSCs), is relatively newer and less invasive with a similar cell differentiation potential. Apart from these two popular sources, Wharton's jelly and dental pulp are the other sources that are being investigated at the preclinical level before human application could be tested. Induced pluripotent stem cells (iPSCs) are another viable option for STE which, on one hand, possess a potency similar to ESCs while, on the other hand, have the advantage of being ethically proven and being an autologous source like mesenchymal stem cells (MSCs). Bilousova et al. demonstrated the differentiation of iPSCs into a functional keratinocyte lineage which further regenerated a fully differentiated epidermis along with hair follicles and sebaceous glands *in vivo* [68]. Gledhill et al. also generated keratinocytes, fibroblasts, and melanocytes from iPSCs further testing their functionality in human 3D skin equivalents [69]. Itoh et al. also generated 3D skin equivalents using iPSC-derived keratinocytes and fibroblasts [70]. However, the transgene technology used to develop iPSCs may lead to carcinogenesis and tumor formation; hence, its use is currently restricted.  Table 1 summarizes the pros and cons of each cell source.

Although all these cell sources have their own advantages, adult stem cells like MSCs have an edge over the others due to their multipotency, wound-healing, and immunomodulatory properties, which make them suitable for allogenic use. Furthermore, they can be sourced out from the adult body and can be banked, eventually overcoming the issues of scarcity and cost.

## 3. Biomaterials for Scaffold Fabrication

Although several novel designs of 3D biological scaffolds to replace the injured skin based on their anatomy and biomechanical and biochemical properties have been proposed, there are several key challenges that still need to addressed. The choice of biomaterial remains the most critical element for any tissue engineering application. For instance, the type and composition of the biomaterial used and its associated properties such as degradation and biocompatibility decides the ultimate fate of the material *in vivo*. Can the scaffold provide the basic structural and biomechanical cues to allow appropriate cellular responses? Can the material properties be easily modulated to support specific cell responses? Can the material be moldable to various geometries such as viscoelastic ink for 3D printing and electrospinning? To address these, scientists are on a quest to create materials (both natural and synthetic) in multimaterial combinations in order to customize each formulation based on their end term application.

### 3.1. Basic Scaffold Characteristics

A scaffold is a temporary 3D structure that facilitates guided growth and differentiation of a functional neotissue by serving as a carrier of cells and other biological factors *via* cell adhesion, migration, proliferation, ECM synthesis, and differentiation (Figure 1) [24, 71]. As discussed before, a scaffold should possess some basic characteristics for it to accomplish tissue repair and regeneration.

#### 3.1.1. Biocompatibility

Biocompatibility stands for the ability to support normal cell activities like cell anchorage, ECM secretion, and cell proliferation without eliciting any type of immunogenic response [72, 73]. For instance, in the case of allogenic grafts, resident cells and ECM proteins prove to be immunogenic. In decellularized grafts, cell remnants like DNA and alpha-gal (a carbohydrate usually found in mammalian cell membrane) serve as common sources of immunogens [74]; however, this issue is prevalent only in the case of xenografts. It is critical to assess scaffolds at both *in vitro* and preclinical levels for screening them against toxicity (fibroinflammatory responses, carcinogenicity). Subcutaneous implantation in animal models is a common way of assessing novel biomaterials for their immunogenicity (validated by the presence of macrophages, neutrophils, and other immune cells) and tissue integration prior to clinical phase trials [75]. Therefore, FDA-approved natural and synthetic biomaterials like collagen [76], silk [77], pluronic F-127 [78], and poly(*ε*-caprolactone) (PCL) [79] are being fabricated alone or in combination to improve their compatibility with the biological tissue. The biocompatibility of a material also depends upon the protein adsorption dynamics on its surface, which influence subsequent cell attachment and proliferation [80]. However, with the large inflow of novel materials, vigorous testing protocols need to be undertaken for evaluating their translational potential.

#### 3.1.2. Biodegradability

Biodegradability refers to the property of the scaffold to degrade naturally in the biological environment at an optimal rate without leaving behind any non-biocompatible by-products. A faster rate of degradation may lead to unsatisfactory mechanical properties and improper tissue regeneration, while slower degradation may increase the chances of fibrotic encapsulation or toxicity [74]. Smart matrices having a tunable degradation rate help mediate the deposition of new ECM at a rate proportional to the neo-tissue formation so that, a fully functional, stable tissue is restored overtime [74]. Optimizing biodegradation is a complex task and is largely influenced by material properties (composition, concentration, geometry, surface area, and processing), method of fabrication, etc. which need to be carefully evaluated in laboratory settings.

#### 3.1.3. Optimal Mechanical Properties

Mechanical properties pertaining to linear elasticity and anisotropy are crucial in scaffold designing for the skin. In the native skin, a dermal ECM comprises of cross-linked fibers of collagen and elastin proteins, which provide the required mechanical framework and elasticity to the tissue [81]. The biomechanical properties of the excised skin tissue in tension studied across various groups have demonstrated a large variability in the range of 2.9-150 MPa [82]. Age is another factor that can significantly contribute towards the mechanical properties of the skin [83]. Despite being constituted of three different anatomical layers with each bearing different mechanical properties, the skin is often mistaken as a homogeneous material in biomedical tests [84], while others have named it as a biphasic system comprising of a more elastic epidermis and the viscoelastic dermis [85]. Most commercial skin replacements only target singular layers for restoring the damaged skin. While not much attention has been given towards optimizing the biomechanical properties of these skin substitutes so far, unsatisfactory mechanotransduction cues to the cultured cells may restrict the proliferative and multilineage potential on these matrices, as documented by various *in vitro* studies [85].

### 3.2. Natural Materials

The use of natural polymers is an important lead in the fabrication of engineered scaffolds. The natural polymers can be either polysaccharides (like chitosan) or proteins (e.g., silk fibroin, collagen, and fibrinogen). Natural materials have the advantage of possessing high cellular affinity and do not face any drawbacks in terms of chronic inflammation, immunological reactions, or toxicity [86].

#### 3.2.1. Silk

**Figure 2:**
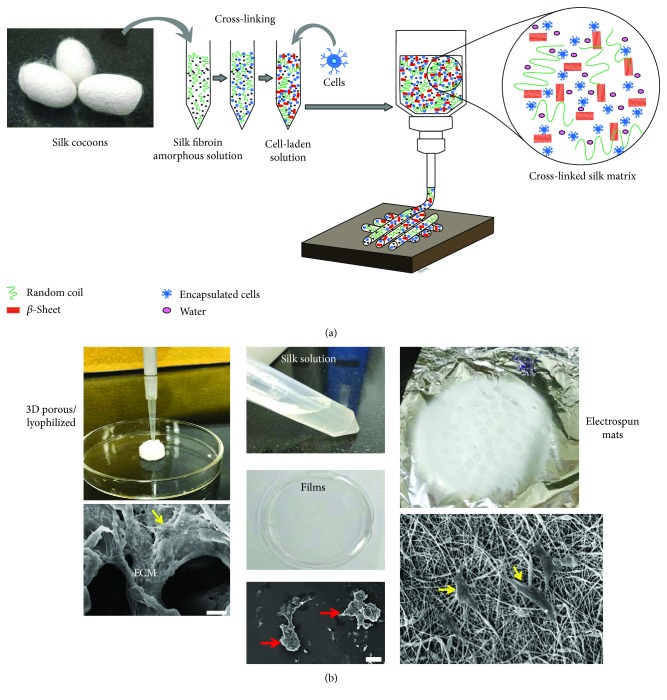
The panel illustrates the processing of silk fibroin solution into various scaffold morphologies by exploiting the physicochemical properties of silk fibroin. (a) Schematic showing the stages of 3D bioprinting: silk fibroin solution is isolated from *Bombyx mori* cocoons in the form of an aqueous solution. The sol to gel transition of this aqueous silk fibroin solution is induced using different cross-linking methods (chemical, physical). Once the rheology is optimized, the silk fibroin hydrogel is mixed with cells and 3D bioprinting is executed under applied pressure (pneumatic or mechanical). (b) Silk fibroin solution is processed in the form of 2D planar films, lyophilized scaffold with 3D porous morphology, and nanofibrous electrospun mats. Scanning electron micrographs demonstrate enhanced cell adhesion, characteristic fibroblastic morphology, and ECM deposition by cultured IHF on 3D scaffolds (lyophilized and electrospun; yellow arrows) as compared to distorted morphology on 2D films (red arrows). Scale bars = 20 *μ*m. Abbreviations: IHF—immortalized human fibroblasts; ECM—extracellular matrix.

Silk has long been used as a dressing for wounds due to its beneficial properties, like good biodegradability, ease of chemical alteration, good oxygen permeability, and the ability of moisture retention [87, 88]. Silk fibroin (SF) is the core protein of the silk fiber derived from cocoons of either mulberry or nonmulberry origin. It consists of a light chain (~26 kDa) and a heavy chain (~390 kDa) which are linked at a ratio of 1 : 1 by a single disulfide bond [89]. The molecular weight of SF has been found to affect wound healing [87]. SF with a narrow range of molecular-weight distribution accelerates healing with better reepithelization, reduced scarring, lower infections and immunogenic responses as compared to SF with a wider molecular-weight range. However, controlling the biomechanical strength of SF-derived scaffolds to match the target tissue properties is a challenging task due to the relatively inferior mechanical properties of regenerated SF [77]. Therefore, the processing of SF from cocoon shells needs to be carefully optimized in order to achieve the desired properties in SF-based constructs. In common lab-based protocols, silk cocoons are dissolved using the standard lithium bromide (LiBr) approach [90], resulting in an aqueous solution of 6 − 8%  *w*/*v* (Figure 2). By exploiting the conformational transition property of silk fibroin from a silk-I to a silk-II structure, the random coils (predominant in the silk-I solution) are cross-linked to aid the transition of sol to gel (with predominant *β*-sheet confirmation), resulting in a hydrogel. This hydrogel form of silk fibroin is heavily exploited in the fabrication of 3D bioprinting approaches (Figure 2(a)). Apart from bioprinting, we have also utilized this property of silk fibroin for the fabrication of constructs in different 3D geometries (Figure 2(b)).

However, several studies have raised concerns with using silk alone in scaffold preparation. These may be related to the clogging of needles in the case of 3D bioprinting and electrospinning (due to the rapid transition into *β*-sheet structure at the time of extrusion), inappropriate mechanical properties due to the length of degumming time [91], and the LiBr dissolution process [90] (which often leads to the degradation of protein chains). In order to circumvent this problem, silk-based composites have been developed for potential applications in different layers of skin such as 3D porous SF functionalized with citrus pectin [92], SF/sodium alginate freeze-dried scaffolds [93], electrospun nanofibers of SF with PLGA [94], SF only [38], collagen-SF [95], and 3D bioprinted SF with keratin [96] and gelatin [97].

#### 3.2.2. Hyaluronic Acid (HA)

HA is a common biological constituent of connective tissues of the cardiac valves, skin, bone, neuronal tissue and umbilical cord. It is an anionic nonsulfated GAG possessing various desirable properties like hydrophilicity, optimal viscoelasticity, and lubrication [98–100]. Being an important element of the vertebrate ECM, HA is nonimmunogenic and provides a congenial environment for cellular growth [101]. This has been observed in recent studies where HA in combination with decellularized porcine ECM and bFGF showed enhanced healing potential in rabbit wounds pertaining to both epidermal and dermal layers [102]. Another bilayered artificial skin substitute composite of HA-gelatin-chitosan demonstrated appropriate mechanical properties and supported the coculture of keratinocytes and fibroblasts for up to 4 weeks *in vitro* [103]. HA can stimulate the production of CD44 receptors during skin healing, which further leads to enzymolysis of HA promoting vascularization and preventing graft contracture. The addition of HA increases the expression of collagens I and III, the primary matrix components of skin [104]. Furthermore, similar to SF, low-molecular-weight HA has been shown to induce fibrovascular tissue growth better than high-molecular-weight HA [105].

#### 3.2.3. Fibrin Glue

Fibrinogen, a glycoprotein in blood, also serves as a potential biomaterial in STE. After its isolation *via* precipitation by ammonium sulfate, PEG, or ethanol, the extracted fibrinogen is converted to fibrin glue (FG) *via* cross-linking by thrombin. The structural and mechanical properties of FG can be controlled by changing the extent of cross-linking [106]. FG possesses many advantageous properties making it a promising choice for STE. Fibrin glue-encapsulated keratinocytes have demonstrated improved healing of burn wounds [27] and leg ulcers [107]. Compared to other naturally derived materials, FG offers a greater versatility in terms of the customization of the skin substitute on the basis of rate of polymerization, geometry, pore size, and fiber thickness by the optimization of some material and physiological properties [107]. Some limitations of FG like high cost, complicated storage conditions, poor mechanical properties, long preparation time, gel shrinkage overtime, and the potential risk of disease transmission limits its applicability in STE applications [28, 108].

#### 3.2.4. Collagen

Collagens are a group of fibrous proteins having a triple-helical structure comprising of *α*-chains. They form the fundamental components of the ECM of almost all the tissues and play an imperative role by aiding in the regulation of tissue remodeling at the time of tissue repair [109–111]. Collagen possesses target motifs for integrin receptors of cells thus regulating various properties related to adhesion, migration, proliferation, and differentiation [112]. Apart from this, easy isolation and purification; reduced toxic levels; and proven chemical, physical, and immunological properties mark its suitability for STE [113]. Encapsulated autologous fibroblasts and keratinocytes in the collagen sponge have been designated as a “true skin substitute,” owing to their capability of promoting faster healing and complete wound closure [114]. Collagen is used in the form of injectable hydrogels, as biocomposites with other polymers for rendering high elasticity, and in the form of films and membranes. However, the weaker mechanical properties and shorter degradation time of collagen resulting from processing parameters have been a big hindrance in the application of this protein. Therefore, alternative strategies have been explored where researchers can incorporate the biological advantages of collagen while overlooking its weaker biomechanical aspect. For instance, collagen scaffolds have been reinforced by combining polymers like PCL which increase the overall tensile strength. Collagen has also been cross-linked by various methods like UV polymerization, glutaraldehyde cross-linking, chitosan blending, and enzymatic treatment to induce various ionic and covalent bonds which improve its mechanical strength. [115]. For instance, glutaraldehyde cross-linking of collagen scaffolds aided in retaining the structural integrity and delaying the process of degradation in skin substitutes when grafted in athymic mice models [116, 117]. While such fixatives acted as good cross-linkers, the residual by-components *in vivo* were found to be cytotoxic [118]. Depending upon the degree of cross-linking induced, collagen-based matrices are degraded by collagenases into peptide fragments and amino acids usually within the time frame of 3–6 weeks, subsequently replacing the scaffold with native type I collagen produced by resident fibroblasts [118]. Therefore, nontoxic cross-linkers such as EDC- (carbodiimide-) NHS (N-hydroxysuccinimide) were applied to collagen structures, which acted as successful potential dermal substitutes *in vivo*. However, the take rate of grafts was compromised in the cross-linked scaffolds over unaltered controls indicating reduced integration of such cross-linked structures. Although, the problem of low take rate was resolved by applying a two-step grafting procedure, it is not the most ideal process for the clinical setting [119]. Hence, more effective and biocompatible methods are being explored such as the use of amino acids L-arginine, glutamic acid, and lysine for bio-cross-linking of collagen which have shown promising outcomes [120].

#### 3.2.5. Decellularized Extracellular Matrix (ECM)

Decellularized ECM scaffolds are widely used in the fabrication of several tissue substitutes in which the donor tissue undergoes removal of cellular components without disturbing the ECM comprising of collagen, GAGs, elastins, and growth factors. Decellularization of the skin allows for complete removal of the resident cell populations (hence reducing immune responses) while retaining the collagen framework, which acts as the reservoir for growth factors and protein components within this ECM network*. In vivo* studies in rat abdominal wall have documented rapid integration of these hydrogels with the host tissue and retainment of the structures for up to 35 days *in vivo* [121]. With the advent of 3D bioprinting, this complex mixture can be explored as a potential bioink (when mixed with autologous cells) to produce substitutes with adequate biological activity for healing cartilage, adipose, cardiac [122], and liver [123] tissues. Acellular dermal matrix (ADM) and acellular amniotic membrane (AAM) have also been used in STE. ADM from the skin of goats, pigs [124], and fish [125] have been used satisfactorily in wound healing. Milan et al. used human decellularized dermal matrix (DDM) seeded with human umbilical cord perivascular cells (HUCPVCs) to treat diabetic wounds in rats [126]. The HUCPVC-loaded DDM scaffolds demonstrated accelerated wound healing and higher VEGFR-2 expression and vascular density than the control groups at 7 days post implantation *in vivo*. However, on one hand as human decellularized ECM has limited availability and is very costly, the use of xenogenic decellularized ECM also exhibits the risk of disease transmission and immunogenicity. AAM serves as an excellent biomaterial for curing the wounds as it aids in pain reduction and moisture retention. Furthermore, it inhibits scarring and extends antimicrobial activity and noninflammatory and antifibroblastic effects [127, 128]. AAMs preserve the tissue ECM properly and have a potential use as a membrane for skin wound healing. TGF-*β*3 expressing bone marrow stromal cells cultured on AAM as a dermal equivalent led to the deposition of parallel, uniform collagen bundles with improved cosmetic appearance and decreased scar formation when transplanted onto full-thickness excisional skin wounds in rats for 85 days [129]. The decellularized matrix can serve as ready-to-use tissue models with essential ECM molecules like collagen, elastins, GAGs, and growth factors for improved cell attachment and proliferation.

### 3.3. Commercial Skin Substitutes

Many commercial skin substitutes are available for skin tissue repair and regeneration. Commercially available skin substitutes can be classified as acellular (e.g., AlloDerm® and Integra®) and cellular grafts. Cellular skin substitutes can be further classified according to the skin layer they are targeting: (a) epidermal (CellSpray, MySkin), (b) dermal (Hyalograft 3D, Dermagraft®), or (c) epidermal-dermal composite (PermaDerm™, Apligraf®) [130]. Griffiths et al. proved that Apligraf® behaves only as a carrier dressing for deep-dermal wounds as the allogenic cells did not survive for long within the matrix in *in vivo* conditions [131]. OrCel™, another cellular skin substitute comprising of fibroblasts laden in bovine collagen type I matrix as the dermal component and keratinocytes seeded at the air-liquid interface as the epidermal component is used commercially for partial-thickness injuries; nonetheless, the use of bovine collagen poses a potential risk of rejection and disease [132]. Similar substitutes like Dermagraft® and TransCyte® are also used in combination with cells. But the long incubation times associated with these substitutes (>6 weeks) may not be favorable for trauma cases [133]. Integra®, the most common commercial skin replacement, was developed in the 1980s by Yannas and Burke as an acellular bilayered construct [134]. It is a porous construct fabricated from bovine tendon collagen and shark GAGs (chondroitin-6-sulfate) which serve as a dermal substitute, while the epidermal representative is the semipermeable polysiloxane (silicone) layer. Most commonly available commercial skin substitutes fall in this category such as Biobrane® and AlloDerm. Biobrane® comprises of a nylon mesh with a silicone membrane mimicking the dermis and epidermis, respectively, in porcine collagen. Though the application involves a single stage procedure, the substitute carries risk of contamination with porcine collagen and is found to be intolerant towards infection sites. While the reported functionality of this substitute is better, the procedure involves a two-stage application hiking the cost of treatment [130]. Commercially available artificial skin substitutes have been widely used in wound-healing studies in combination with both autologous and allogeneic skin cells. A full-thickness skin substitute developed for foot ulcers is Tiscover™. However, these products bear their own limitations, and as such there is no ideal skin substitute yet. No construct so far has been able to recapitulate the 3D geometry, chemistry, and functionality mimicking the native skin tissue. They face several limitations like nonintegrity, immune rejection, poor take, and reduced mechanical strength [130]. Therefore, a tissue-engineered construct with off-the-shelf availability is urgently needed for large-scale application.

## 4. Current Innovative Strategies in Skin Tissue Engineering (STE)

Although great progress has been made in reducing morbidity and mortality occurring as a result of burn wounds, some of the most exciting advances remain ahead. Tissue-engineered skin substitutes using a combination of scaffolds and growth factors appear to be a promising alternative. Use of multimaterial strategy to develop composite scaffolds helps alleviate the limitations associated with individual materials such as inferior mechanical properties and biocompatibility [135]. The ultimate aim is to completely restore skin anatomy and physiology using (a) advanced nanofunctionalized materials for triggering specific responses using nanotechnology; (b) automated and robotic fabrication of engineered tissues to increase efficacy, reduce costs, and cater to individual patient needs using 3D bioprinting; and (c) regenerative therapy using stem cell technology for individually targeting pigmentation, wound closure, angiogenesis, and skin sensation.

### 4.1. Nanotechnology

Nanotechnology has been used in two ways in STE; firstly, in scaffold fabrication and secondly, in loading the scaffolds with growth factors and/or drugs for targeted delivery to the tissue. Electrospinning is a widely used technique for scaffold fabrication in which the polymer solution is spun into nanofibers under the force of an electric field. Advantages of electrospun scaffolds are large surface-to-volume ratio, better pore interconnectivity, easy reproducibility, and easy fabrication method. The nanofibrous architecture aptly mimics the ultrastructure of native tissue ECM; hence, it strengthens the resultant cell-matrix interactions *in vitro* [136]. PLGA electrospun scaffolds have shown to promote fibroblast survival and maintenance in *in vitro* culture for 5 days [137]. Using this material, researchers could achieve a pore size of 100-200 *μ*m for the culture of fibroblasts towards skin tissue engineering applications [138]. On the contrary, Chen et al. successfully demonstrated infiltration of human dermal fibroblasts and subsequent collagen type I synthesis after 7 days on PLGA electrospun scaffolds possessing pore sizes as low as 5-40 *μ*m [139]. In another study, electrospun scaffolds made of collagen showed enhanced cell growth and organization, significantly reducing wound contraction by 22% in full-thickness wounds created in murine models as compared to freeze-dried scaffolds [140]. This is mainly attributed to the increased surface area, interconnectivity, and pore size of electrospun scaffolds over their freeze-dried counterparts. Park et al. used salt (NaCl) crystals within the silk-polyethylene oxide (PEO) electrospun fibers [38]. The resultant scaffolds had pore sizes (250-300 *μ*m) large enough to support the formation of two-tiered skin *in vitro*. An aqueous-based fabrication strategy allows for easy inclusion of biological moieties at the time of electrospinning procedure [75]. Arg-Gly-Asp- (RGD-) functionalized and protease-sensitive poly(ethylene glycol) (PEG) hydrogels have been developed [141]. The addition of cell adhesive and protease-sensitive peptides is very useful as they allow increased cellular attachment, growth, and migration on the matrix.

Another effective alternative is the incorporation of nanoparticles into the engineered scaffolds for targeting specific functions, for instance, excessive fluid loss and on-site infections experienced by chronic-burn patients. Also, exudates from the wounds are a common cause of infections resulting in acute inflammation which pose a hindrance in wound healing. To circumvent this, a collagen–chitosan-based scaffold with silver nanoparticles was developed for repairing the dermal layer of the skin. The silver nanoparticles exhibited bactericidal properties with enhanced biocompatibility of the construct [142]. Another group fabricated electrospun collagen-PEO nanofibrous scaffolds incorporated with gold nanoparticles [143]. Gold nanoparticles are known to enhance biocompatibility and mechanical strength and provide antioxidation and enzyme-resistance to the constructs. All these parameters were tested across a range of concentrations, with 14.27 ppm marked as the most optimal concentration of gold nanoparticles for collagen-PEO electrospun mats. The scaffolds demonstrated negligible toxicity and sustained the culture of murine 3T3 fibroblasts and keratinocytes up to 14 days *in vitro.* The technique of nanoparticle formation has been extended to drug delivery in wounds leading to better healing response. Nanoparticles have several advantages over use of scaffolds. For instance, particles can be injected to the healthy tissue around the wound, preventing any direct manipulations with the wound bed [144]. They can be precisely modified to regulate the required release profiles *in vivo* to match the physiological body conditions [145]. PLGA is the most common polymer used to develop nanoparticles [146]. Chereddy et al. developed curcumin-loaded PLGA nanoparticles which, along with lactate, showed enhanced angiogenesis and reduced inflammatory response in full-thickness splinted excisional wounds in mice [147]. In another unique approach, silica, PEG, and chitosan were mixed to develop nanoparticles for sustained release of nitric oxide in wounds accelerating the healing process in infected, noninfected, and diabetic wounds in mice models [144, 148, 149]. In yet another approach, lyophilized keratinocyte-targeted nanocarriers loaded with locked nucleic acid- (LNA-) modified anti-microRNAs (miRs) were applied increasing the expression of Dicer which plays a pivotal role in reestablishing the barrier function of the skin [150]. Hence, nanotechnology has a wide-ranged application in STE.

### 4.2. Scaffold-Free Approach

Scaffold-free approach involves the use of a carrier-free or matrix-free cell population cultured in the form of a transplantable cell sheet for direct implantation onto the site of injury. The technique is useful in avoiding the immunogenicity associated with most synthetic scaffolds. However, the lack of a mechanical support fails to provide anchorage to the proliferating cells from the host. Thermoresponsive materials such as poly(N-isopropylacrylamide) may provide an effective carrier-free sheet. It was observed that this thermosensitive property of the polymer could be applied to modulate changes in the scaffold's pore diameter, i.e., the pore size decreased with an increase in temperature [151]. A porous scaffold of poly(N-isopropylacrylamide) developed using the sphere-templating technique with a pore diameter of 55 ± 5 *μ*m was cultured with NIH3T3 fibroblasts, and the temperature was subsequently changed to 37 °C. The resulting phase transition constricted the pore size to 39 *μ*m, which is the optimal diameter for facilitating angiogenesis and matrix synthesis. These cells were cultured for 7 days *in vitro* and showed a characteristic elongated morphology. Liu et al. constructed a scaffold-free bilayered, vascularized tissue-engineered skin by superimposing 4 layers of dermal fibroblasts and endothelial cell sheets to form the dermal layer followed by seeding and culture of keratinocytes on it. The sustainability of this *in vitro*-developed vascularized skin containing epithelial cells, endothelial cells, and fibroblasts was up to 5 weeks in culture [152]. Recently, the scaffold-free approach has also been applied to 3D bioprinting. A novel bioink formulation comprising of 10%  *w*/*v* gelatin, 0.5%  *w*/*v* alginate, and 2%  *w*/*v* fibrinogen was 3D bioprinted with human dermal fibroblasts to develop a construct of 1 cm × 1 cm × 0.5 cm dimensions. The study could successfully demonstrate a printable, clinically conformant functional skin tissue characterized at the molecular level [153].

### 4.3. Stem Cell Technology

Recent advances in stem cell-based therapeutics have propelled an increasingly high enthusiasm in STE. The essential ingredients for successful STE include the choice of biomaterials combined with the appropriate cells and growth-inducing factors [154]. Different types of stem cells have been explored in the field of STE.

#### 4.3.1. Mesenchymal Stem Cells

Mesenchymal stem cells (MSCs) are multipotent cells found in adults in various tissues like adipose, bone marrow, and dental pulp. BMSCs have been shown to promote angiogenesis, epithelialization, granulation, and tissue formation in an *in vivo* setup leading to effective skin regeneration [155]. BMSCs cultured on collagen matrices have shown therapeutic potential across a variety of wounds [156, 157]. Furthermore, BMSC-derived exosomes are being investigated as mediators in wound healing [158].

ADSCs have also been widely used in wound healing studies. When encapsulated in fibrin-chitosan hydrogel matrices, they have demonstrated a consistent release of angiogenic factors to aid in the wound healing process [159]. Conditioned media of ADSCs stimulated collagen deposition and homing of human dermal fibroblasts [160], thus establishing the role of ADSCs in accelerated wound repair *via* the secretion of growth factors. Release of angiogenic cytokines by ADSCs augmented the degree of neovascularization [161]. A more recent vascularization strategy involves the use of ADSC-derived microvascular fragments isolated from mice fat pads [162]. These microvascular fragments seeded onto collagen-GAG biomatrices implanted into the dorsal skinfold chambers of C57BL/6 mice showed dense microvascular branching and lymphatic networks after 14 days. On the contrary, this angiogenic response of ADSC-derived microvascular fragments was rather diminished when porous scaffolds were precultivated with microvascular fragments for 28 days *in vitro* and then subjected to the *in vivo* dorsal chamber in mice [163]. This study highlighted the importance of freshly isolated microvascular fragments from mice fat pads for *in vivo* implantation purposes. A successful application of these ADSC-derived microvascular fragments was also recently documented in a full-thickness skin wound model by significantly improving the vascular and lymphatic networks when applied together with STSG [51]. Chan et al. showed the effect of different hydrogels on the multilineage differentiation potential of ADSCs [164]. Lin et al. determined that application of ADSC sheets accelerates the rate of tissue healing by as early as 18 days post treatment in comparison to the control group [165]. Trottier et al. established that ADSCs could serve as a plausible alternative for fibroblasts in STE by fabricating a trilayered skin substitute using ADSCs only [166]. Gholipourmalekabadi et al. combined stem cells, nanotechnology, and the healing properties of the amniotic membrane to develop a 3D bilayer scaffold for burn injuries [167]. In the study, silk fibroin was directly electrospun on decellularized amniotic membrane to develop the bilayered scaffold. Postfabrication, ADSCs were cultured on the scaffold for 15 days which resulted in increased expression of two proangiogenic factors, vascular endothelial growth factor (VEGF) and bFGF, which are prerequisites in wound healing and hence determine the efficiency of the skin substitute. Another potential source of stem cells, human dermal papilla, isolated from the dental pulp have shown promising potential in hair follicle regeneration by self-organizing into *in vitro* organoids [168].

It is a challenge to heal larger wounds and ulcers that can only be treated with surgical grafts so far. Recent research has shown successful reprogramming of wound-resident mesenchymal cells in *in vivo* mice models which were able to regenerate skin epithelial tissue [169]. Cellular reprogramming *in vivo* was conducted by injecting viruses to induce the expression of four specific genes in the cells of nonhealing ulcers to transform them into epithelial cells. These mice were able to demonstrate complete healing of the large wounds by a newly formed layer of epithelial cells within 28 days. This novel strategy provided an effective solution towards surgical skin transplants or artificial skin grafts for hard-to-heal wounds, particularly in aged population and diabetic people [169].

#### 4.3.2. Skin Stem Cells

Stem cells in the skin mainly reside in the hair follicles, sweat glands, and stratum basale of the epidermis. These alternate sources of stem cells have also been explored for STE applications. In one of the approaches, human stem cells derived from sweat glands when seeded onto Integra® (a commercial skin replacement possessing collagen fibers cross-linked with GAGs) demonstrated enhanced dermal regeneration by increasing the extent of vascularization in a bilateral full-skin wound. The stem cells were homogenously distributed on the scaffold exhibiting satisfactory cell-substrate interactions [170]. Nestin-positive stem cells have also been known to contribute towards wound healing *via* the development of intricate microvasculature networks [171, 172]. Induction of neovascularization by nestin-expressing hair follicle cells that successfully anastomosed with the host vasculature was demonstrated by Amoh et al. in nude mice models [171]. According to a clinical study, a team of medical practitioners treated a 7-year-old boy suffering from epidermolysis bullosa, a genetic skin disease also called butterfly disease, wherein approximately 80% of the epidermis was damaged. Scientists attempted to regenerate the damaged skin using transplants derived from modified stem cells. Procedurally, the autologous epidermal stem cells were isolated from the patient and the gene defect was repaired by adding a corrected form of the mutated gene. Such patient-derived cells containing the genetically corrected gene were grown in a laboratory in order to form a sheet of neoskin tissue. Nearly 400 million of such genetically corrected cells were grafted into the patient, but most of the transplanted cells died after the procedure. Of the transplanted cells, the relatively stable epidermal stem cells were found to contribute towards the generation of the new skin tissue. After a month, the patient showed successful signs of skin restoration in the affected area. Furthermore, after 2 years of follow-up, the newly regenerated skin showed molecular markers characteristic of morphologically stable and functional skin tissue. Thus, the combined approach of cell and gene therapy could successfully regenerate approximately 80% of the patient's skin [173].

#### 4.3.3. Embryo-Related Stem Cells

Embryo-related stem cells include the embryonic stem cells and stem cells from extraembryonic tissues like the placenta, umbilical cord, and amniotic fluid. Stem cells from these sources have a greater potency than adult stem cells. Skardal et al. used laser deposition bioprinting to print amniotic-fluid stem cells on dorsal skin wounds in mice to investigate their effect on wound healing [174]. Amniotic-fluid stem cells were selected owing to their tremendous proliferative ability, enhanced angiogenic potential, and nonimmunogenicity. Immunohistochemistry showed that stem cells left the wound site by day 7 suggesting that the stem cells contributed towards the early days of healing by migration, drastic release of growth factors, and their propensity to maintain an undifferentiated state for extended periods leading to increased cell proliferation [174].

Isolating a differentiated population of autologous cells becomes complicated in cases where extensive injury or burns are inflicted covering a large body surface area. Hence, stem cells are a rescue in such scenarios as they possess the tremendous potential of self-renewal and multilineage differentiation, so that the scientists can start with low initial cell density [154].

Another critical parameter is the damaged sweat glands in burn victims as well as in some genetic disorders, which can be a life-threatening condition. Such people are unable to exercise properly as it may lead to heat stroke and brain damage. Hence, the creation of sweat glands using skin grafting procedures is clinically important for the regeneration of a viable skin model. Current clinical methods of skin grafting and regenerative therapy are incapable of inducing the development of functional sweat glands. Recent research in the field has highlighted the relevance of precise control in the development of sweat glands at the molecular level. The signaling pathways responsible for the development of hair follicles and sweat glands are similar but express at different time frames. The signaling cascade inducing the expression of hair follicles is triggered first, followed by the expression of bone morphogenetic proteins (BMPs), which helps to create sweat glands. This deep understanding into the underlying signaling mechanisms can prove to be very beneficial in the way of improvising on the skin tissue engineering methods for effective skin grafting procedures [175]. For instance, the biomolecular cues from fibroblast growth factor-2 (FGF2) and vascular endothelial growth factor- (VEGF-) loaded StarPEG-heparin hydrogel triggered differential cellular behavior of human umbilical vein endothelial cells (HUVECs) indicating proangiogenic conditions [176]. Similarly, Zhou et al. showed that downregulating the expression of microRNA-203 at the wound site by the application of antimicroRNA-203 can accelerate wound healing and reduce scarring. Downregulation of microRNA-203 increases the expression of the gene Hairy/Enhancer of split-1 (Hes1) which is a downstream signaling molecule in the Notch1/Jagged1 pathway which in turn promotes epidermal stem cell proliferation and inhibits its differentiation [177].

However, for over several decades, human skin cells have been cultured in combination with animal culture systems, which create potential risk of infections and immune complications. To overcome these limitations, researchers developed a fully animal-free, human-based system for the cultivation of skin cells for safer skin grafting [178]. The researchers used laminin proteins, specifically LN-511 or LN-421, as animal-free components for providing a robust yet safe *in vitro* cell culture system for effective skin grafting. This may lead to a more progressive research approach by pushing such *in vitro* standardized systems towards clinical application.

### 4.4. 3D Bioprinting

**Figure 3:**
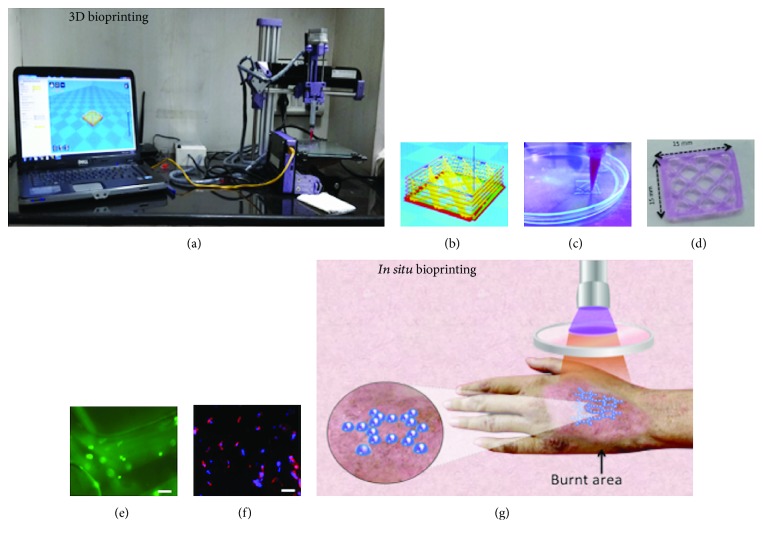
3D bioprinting to develop custom-made skin tissue constructs. (a) A customized printer used in our lab (by Alfatek Systems, Kolkata). (b) The CAD image prepared using the inbuilt software in a readable file format for bioprinting. (c) Following the CAD image, a pluronic-based bioink mixed with fibroblasts is loaded into the syringe fitted with a nozzle and the printing process is executed under applied pressure. (d) 3D bioprinted construct. (e) Fluorescence micrograph of GFP-tagged fibroblasts (green) showing cellular distribution inside the filaments of the construct immediately after printing. (f) Protein expression of skin-specific marker vimentin (red) in a 3D bioprinted pluronic-based construct 3 days postprinting by immunofluorescent staining. Nuclear staining was done by DAPI (blue). (g) *In situ* bioprinting strategy schematically depicted on the burnt skin of a patient demonstrating deposition of the bioink directly on the region of interest. Scale bars = 30 *μ*m. Abbreviations: CAD—computer-aided design; IHF—immortalized human fibroblasts. Immortalized human fibroblasts (IHF) were used for 3D bioprinting.

3D bioprinting is a computer-aided advanced technology involving the precise placement of cells into predetermined 3D patterns. At the preclinical level, the technique has showcased successful replication of natural skin anatomy [179] as compared to conventional fabrication methods employing top-seeding of cells [180]. 3D bioprinted scaffolds are fabricated using the following two approaches: (a) top-down approach, in which cells are directly made available to a prefabricated biomimetic scaffold for tissue maturation in a bioreactor and (b) bottom-up approach, which provides only an initial temporary support and depends on the seeded cells for the deposition of ECM [181, 182]. For bioprinting, the biomaterial processed in the form of “ink” should also be “printable” apart from being biocompatible and durable. Printability depends on two attributes: rheology (covers aspects like shear thinning and viscoelasticity of material) and cross-linking methods employed (chemical (enzymatic), physical (sonication), photodependent, temperature-dependent, or ionic mechanism) [183]. The inks are usually made from synthetic polymers, like PCL, PLGA, PEG, and pluronic F-127, or natural polymers, like silk fibroin, collagen, or fibrinogen [63] or an amalgamation of the two types of materials. Apart from the material or ink, the optimal choice of cells is another critical component in fabricating constructs. The most commonly used cells for skin constructs have been keratinocytes and fibroblasts [38]. Using a customized 3D bioprinter built by Alfatek Systems, Kolkata, we have successfully fabricated custom-made 3D bioprinted constructs made of pluronic-based bioink mixed with immortalized human fibroblasts (IHF). The constructs demonstrated optimal viscosity for extrusion bioprinting and post-printing stability by retaining the multilayered stack of the construct. In addition, the green fluorescent protein- (GFP) tagged IHF population was viable for more than 7 days in *in vitro* culture and showed characteristic fibroblastic markers, as shown in Figure 3.

Lee et al. in 2009 were the first to successfully generate a 3D bioprinted human skin construct using collagen type I with a co-culture of fibroblasts and keratinocytes [63]. A stratified construct was printed by sandwiching the collagen matrix between fibroblasts and keratinocytes. The printed cells showcased characteristic cellular morphology; however, the proliferation was affected by poor printing resolution, which was far from the optimal resolution of 300 *μ*m. In another landmark study, Binder was the first to demonstrate *in situ* bioprinting of a dorsal skin defect (10 × 10 cm) in a porcine model [184]. The wound dimensions were measured by laser scanning, and the bioink made from collagen-fibrinogen with keratinocytes and fibroblasts was delivered using an ink-jet bioprinter onto the wound. Complete reepithelialization occurred by the 8th week, which was significantly enhanced as compared to all the control groups (allogenic, untreated, and hydrogel matrix only). The concept of *in situ* bioprinting comes from the fact that the injury/lesion is repaired by bioprinting the cell-material biocomposite directly on the target site (Figure 3). Advantages include rapid implantation, no delay in surgical time, and reduced risk of on-site infections. Another form of bioprinting called laser-assisted bioprinting, which exploits laser-induced forward deposition, was used by Koch et al. [185] for STE. The procedure involved depositing the cells onto a MatriDerm® scaffold (a commercial graft made up of collagen and elastin) using a laser pulse inducing cell knockouts with high precision from a laser-absorbing donor layer (~60 nm in thickness) directly onto the scaffold. Cadherins, the molecules forming the gap junctions, were observed at the basal lamina (Cx43 expression) indicating differentiation and maturation of the printed tissue. Michael et al. also used laser-assisted bioprinting to fabricate a bilayered skin construct made of collagen comprising of the primary constituent skin cells (fibroblasts and keratinocytes) and demonstrated full-thickness wound healing in mice [179]. Ki67 staining confirmed the presence of the proliferative layer (stratum basale) of the skin. Inadequate tissue differentiation and vascularization were observed at the site of implantation, which may be attributed to the short experimental duration of only 11 days.

Recently, Lee et al. used a robotic approach in a solid freeform fabrication system with contactless dispensers capable of achieving a resolution of ~500 *μ*m (approximately 28 nl of the bioink) [63]. A density of 2.3 × 10^6^ cells/ml was used to print both keratinocytes and fibroblasts. More recently, Cubo et al. printed a biocomposite of skin cells mixed with human plasma using an in-house modified extrusion bioprinter having four dispensers [39]. The bioprinted product was prepared on a P100 plate before grafting it subcutaneously in an immunodeficient mice for 8 weeks, which is one of the maximal time periods tested so far *in vivo*. At the end of 8 weeks, the implanted skin exhibited the generation of two new layers, the basal lamina and the stratum corneum, indicating complete differentiation. The biggest achievement of this study was the ability to bioprint skin constructs in <35 minutes highlighting the quick application and translational potential of the technology.

Next, advances in 3D bioprinting have led to the development of constructs with a controlled pore architecture and guided cellular assembly *in vitro* for the synthesis of sweat gland mimic [186]. The drop-on-demand technology can help place the cells or the polymer precisely in a spatiotemporal pattern so that the complex structures like pigmentation unit, hair follicle, or sweat gland can be fabricated as required. However, the technique needs to be extensively standardized in terms of the matrix with an adequate mechano- and compositional niche for cell proliferation, migration, and morphogenesis for generating phenotypically stable and complex structures. While it is clear that fibroblasts, keratinocytes, and stem cells are printable using various inks, but reproducing the integrity and functionality of the human skin for STE applications and inducing sensation have not been achieved yet. Some of the challenges being faced include printing resolution, adequate vascularization, choice of optimal cell type and source, bioink composition to match the zonal tissue architecture, growth factor gradient, dimensions of clinically conformant constructs, cost of bioprinted skin, and the complexity of the technique before clinical success could be envisaged. Scalability, although today a challenge, can be overcome in the very near future with fastly evolving software and machinery, which require relatively lower initial cell densities. This helps in meeting the demands for developing constructs to cover a large body surface; a major limiting factor with the autologous grafts; current gold standards. With the evolving field of 3D bioprinting, scalability can be combined with customization of the graft, achieving a major milestone in STE and wound healing. Smaller 3D skin tissue *in vitro* models are at a better position of being translated for applicability in *ex vivo* testing of cosmetics and drugs, before this technology reaches the hospitals for reconstructive surgeries [187]. The success of the technology in the research field is reflected by cumulative investments of > $44 million in grants made by companies like Procter & Gamble, L'Oréal, and Poietis into R&D of 3D bioprinted functional *in vitro* skin models [187]. However, abundant work needs to be undertaken for determining the underlying molecular mechanisms that regulate tissue differentiation in such artificial *in vitro* model systems for better exploitation of the technology.

## 5. Future Prospectives

The field of STE is maturing and the biomaterials used (such as collagen) in developing products have benefited patients over decades. However, even after decades of research in the field of STE, production of large constructs for victims with more than 50% skin loss is still a challenging proposition. Despite the availability of a large number of techniques and commercial skin substitutes, the quest for a functional artificial skin capable of supporting skin functions like thermoregulation, sensation, perspiration, and UV radiation protection with normal aesthetic appearance is still on [130]. Several biomaterials supplied as commercial skin substitutes are available, albeit with associated limitations. For instance, risk of disease transmission from the use of porcine collagen could be prevented using synthetic collagen-based materials or recombinantly produced collagen. Next task would be to identify the cell adhesion and tissue integration properties of this synthesized collagen. Also, such cell-free biomaterials may act as appropriate carriers/substrates for smaller injuries where host cells could migrate into the wound site. With larger wounds, the biomaterials alone are not sufficient. They need a prevascularized bed with cells to trigger the regenerative process. To achieve these functions, stem cell-based tissue engineering therapy has grasped much attention owing to their self-renewal capacity and multilineage potential, critical for mimicking the physiological hierarchy and functions of native tissue. However, finding the optimal cell source, standardization of processing, and application in clinical settings, as well as precisely regulating the response of stem cells *in situ*, is still subjected to consideration. The other major problem is with scalability of technique. Most of the research has been carried out on small wounds created in rodent models; therefore, critical measures need to be undertaken to escalate their applicability for preparing clinically conformant skin grafts [2]. The robustness and reproducibility of nanotechnology-based techniques make it suitable for producing off-the-shelf products while, on the other hand, an advanced technique like 3D bioprinting is capable of developing customized substitutes. Each of the approaches has its own lacunae which can be overcome by permutating and combining the available techniques so as to develop a skin substitute which can meet the needs of the wound care market. Therefore, there is a pressing demand for more active and high-paced research in order to cope with the increasing demand for skin tissue grafts. With the advent of cutting-edge technologies, fabricating a functional patient-specific skin substitute does not appear to be far-fetched.

## Figures and Tables

**Table 1:** Advantages and disadvantages of various cell sources used in STE.

Cell source	Advantages	Disadvantages
Embryonic stem cells	Pluripotent Abundant source	Ethical concerns Tendency to form teratomas
Induced pluripotent stem cells	Pluripotent Ethically approved Autologous	Difficult to develop Carcinogenic and tumorigenic tendency
Differentiated primary cells	Closest to in vivo Autologous	Difficult to isolate and maintain
Adult stem cells		
(1) Bone marrow-derived mesenchymal stem cells	Clinically tested Can be used in allogenic setting	Invasive procedure of isolation
(2) Adipose-derived stem cells	Non-invasive procedure of isolation Can be used in allogenic setting	Clinically unapproved
(3) Skin stem cells	Predisposed to differentiation into skin cell lineage	Difficult to isolate
Cell lines	Robust; easy to culture	Genetically altered

## Abbreviations

3D: Three dimensional
AAM: Acellular amniotic membrane
ADM: Acellular dermal matrix
ADSCs: Adipose-derived stem cells
bFGF: Basic fibroblast growth factor
BM-MSCs: Bone marrow-derived mesenchymal stem cells
BMSCs: Bone marrow stem cells
CAD: Computer-aided design
CEA: Cultured epithelial autografts
Cx43: Connexin 43
DDM: Decellularized dermal matrix
DNA: Deoxyribonucleic acid
ECM: Extracellular matrix
ESCs: Embryonic stem cells
FDA: Food and Drug Administration
GAGs: Glycosaminoglycans
GFP: Green fluorescent protein
HA: Hyaluronic acid
IHF: Immortalized human fibroblasts
iPSCs: Induced pluripotent stem cells
LiBr: Lithium bromide
MSCs: Mesenchymal stem cells
NaCl: Sodium chloride
PCL: Poly(*ε*-caprolactone)
PEG: Polyethylene glycol
PEO: Polyethylene oxide
PLGA: Poly(lactic-co-glycolic) acid
RGD: Arginine-glycine-aspartic acid
SF: Silk fibroin
STE: Skin tissue engineering
STSG: Split-thickness skin grafts
USD: United States dollar
UV: Ultraviolet
VEGF: Vascular endothelial growth factor
WHO: World Health Organization.
